# A hierarchical inventory of the world’s mountains for global comparative mountain science

**DOI:** 10.1038/s41597-022-01256-y

**Published:** 2022-04-01

**Authors:** Mark A. Snethlage, Jonas Geschke, Ajay Ranipeta, Walter Jetz, Nigel G. Yoccoz, Christian Körner, Eva M. Spehn, Markus Fischer, Davnah Urbach

**Affiliations:** 1 Global Mountain Biodiversity Assessment (GMBA), University of Bern, Bern, Switzerland; 2 Institute of Plant Sciences, University of Bern, Bern, Switzerland; 3 School for Forestry and the Environment, Yale University, New Haven, United States; 4 Department of Ecology and Evolutionary Biology, Yale University, New Haven, United States; 5 Department of Arctic and Marine Biology, UiT The Arctic University of Norway, Tromsø, Norway; 6 Department of Environmental Sciences, Botany, University of Basel, Basel, Switzerland; 7 Swiss Academy of Sciences (SCNAT), Bern, Switzerland

**Keywords:** Biodiversity, Biogeography, Biodiversity

## Abstract

A standardized delineation of the world’s mountains has many applications in research, education, and the science-policy interface. Here we provide a new inventory of 8616 mountain ranges developed under the auspices of the Global Mountain Biodiversity Assessment (GMBA). Building on an earlier compilation, the presented geospatial database uses a further advanced and generalized mountain definition and a semi-automated method to enable globally standardized, transparent delineations of mountain ranges worldwide. The inventory is presented on EarthEnv at various hierarchical levels and allows users to select their preferred level of regional aggregation from continents to small subranges according to their needs and the scale of their analyses. The clearly defined, globally consistent and hierarchical nature of the presented mountain inventory offers a standardized resource for referencing and addressing mountains across basic and applied natural as well as social sciences and a range of other uses in science communication and education.

## Abstract (summary)

**Table:** 

Measurement(s)	mountain range
Technology Type(s)	Geographic Information System
Factor Type(s)	ruggedness
Sample Characteristic - Environment	mountain
Sample Characteristic - Location	global

## Background & Summary

In recent years, the number of scientific reports, syntheses, assessments, and cross-scale comparisons of patterns and trends in natural and social systems has increased rapidly. These efforts represent a wealth of knowledge and unique resources for agenda setting and negotiations. However, a growing challenge inherent to these contributions lies in the partitioning of the world into relevant and comparable (mapping) units for data aggregation, analysis, and reporting. Different means of regionalization are adopted for various purposes and in different fields. General purpose approaches include administrative units^1^, watersheds or river basins^2^, landforms^3^, ecoregions^4^, terrestrial habitats^5^, or the GEO, IPBES or IPCC (sub-) regions and units of analysis. Examples of more specific partitioning schemes include the World Geographical Scheme for Recording Plant Distributions^6^. Any layer that shows the location and extent of geographical features allows the detailed spatial localization of field data^7^ and their analysis at different resolutions. However, the use of different regionalizations also leads to different results, which calls for a clear understanding of the spatial geometry and extent of mapping units and a well-founded rationale for their adoption^8^.

In mountains, the integrative and comparative biogeographical and socio-ecological sciences needed for global reporting (e.g., Aichi target 14 on the restoration and safeguard of essential services, SDG target 15.4 on the conservation of mountain ecosystems) and safeguard have long been hampered by a lack of consensus about the definition of mountains^9–12^. Indeed, assigning an outer border to mountainous regions always remains arbitrary as mountain ranges are inherently fuzzy physiographical objects that often gradually merge into the surrounding terrain^13^, and because there is a considerable amount of (personal, cultural, and regional) subjectiveness in deciding what a mountain is^9^. This long-standing lack of consensus, which accounts for an approximate 60% difference between estimates of global mountain coverage^8,10,12^, served until recently as an explanation for the absence of a global inventory of the world’s mountains. In 2017, Körner and colleagues filled this gap with the first of its kind global mountain inventory (GMBA Inventory v1.0)^10,14^. This inventory was based on a definition of mountains proposed by Körner *et al*. in 2011^15^ (GMBA Definition v1.0) and consisted of 1003 hand-drawn shapes (GIS vector polygons) representing mountain ranges. An updated version including 1047 shapes (v1.4) was released in 2019.

**Fig. 1:**
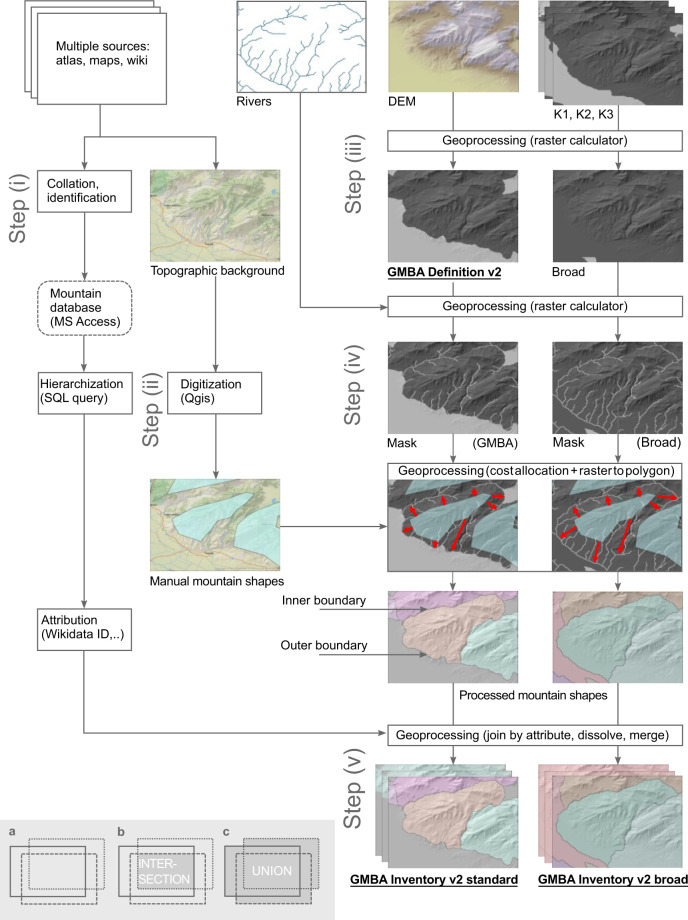
Simplified workflow for creating the GMBA Mountain Inventory v2.0. Bold and underlined map names represent final products. Inset: Combinations of the different mountain delineations used in this paper. Full, dashed and dotted rectangles represent each of the three delineations (K1, K2 and K3). The grey area in (**b**) represents the intersection of the three layers (Boolean operator ‘AND’) which was used as a training area for determining the elevation range thresholds for each NAW (step iii, see also Fig. 2), while (**c**) represents the geometric union of the three layers (Boolean operator ‘OR’), which was used in creating the ‘broad’ mountain delineation layer.

Here we propose a new inventory of the world’s mountains based on a refined mountain definition. Our new inventory delimits 8616 mountain ranges and overcomes several of the limitations of the first one by introducing a hierarchical structure and using rivers to establish the borders between contiguous mountain ranges (‘inner borders’, Fig. 1). This ensures global consistency in the level of detail and resolution, and a high accuracy. The hierarchical structure allows customized aggregation into specific units of analysis for global mountain sciences, policy, advocacy, and collective action^16,17^. Our refined mountain definition, in turn, distinguishes itself from existing ones by relying on a set of parameters derived directly from digital elevation models (DEM), and not on expert judgement.

We developed interactive visualizations of the resource on the GMBA mountain inventory page (https://www.earthenv.org/mountains)^18^ of EarthEnv to support a versatile exploration of potential uses and download the shapefiles. The dedicated EarthEnv app currently allows a choice of preferred hierarchical level and provides basic statistics. Further tools built on top of this initial browser are in development. Additionally, a set of R functions is provided on Github (https://github.com/GMBA-biodiversity/gmbaR) to work with the inventory.

Applications for which this inventory is particularly well-suited include the geolocation of data and knowledge pertaining to specific mountain ranges (e.g. species occurrence and distribution), the extraction of data from spatial layers (e.g. human population and settlements) at the level of named mountain ranges, as well as the spatially-explicit hierarchical aggregation of data for analysis of and reporting on patterns in mountain social-ecological systems.

The reference to, or application of, the GMBA Inventory v1.0 in more than 90 studies across the natural and social sciences (See https://www.gmba.unibe.ch/services/tools/mountain_inventory, for a list of publications) and its uptake on several online platforms attest to the usefulness of such a standardized tool. Its application also served to build a community of users whose feedbacks informed the development of the inventory we present here. By offering the flexibility for both the aggregation and the disaggregation of data and knowledge at different scales, our new inventory greatly expands the scope of potential applications of such a tool.

## Methods

The generation of this map of the world’s mountains consisted of five steps (Fig. 1): (i) the identification and hierarchisation of named mountain ranges and the recording of range-specific information; (ii) the manual digitization of the ranges’ general shape; (iii) the definition of mountainous terrain (and the inventory’s outer borders) using a DEM-based algorithm; (iv) the automatic refinement of the digitized and named ranges’ inner borders; and (v) the preparation of the final layers. The resulting products consist of a refined mountain definition (*GMBA Definition v2.0*), two versions of the inventory (*GMBA Inventory v2.0_standard* & *GMBA Inventory v2.0_broad*), and a set of tools to work with the inventories.

### Step i: Identification and hierarchisation of mountain ranges

In a first step, we identified mountain ranges worldwide. To do so we adopted the mountain ranges identified in the GMBA Inventory v1.4^10,14^ and searched existing resources in any languages for other named ranges not yet included. The ranges added could either be adjacent to, included in (child range or subrange) or including (parent range or mountain system) mountain ranges of the GMBA Inventory v1.4. The resources used for our searches included world atlases (e.g. The Times Comprehensive Atlas of the World^19^, Knaurs grosser Weltatlas^20^, Pergamon World Atlas^21^); topographic maps (e.g. http://legacy.lib.utexas.edu/maps/imw/, http://legacy.lib.utexas.edu/maps/onc/, https://maps.lib.utexas.edu/maps/tpc/, www.topomap.co.nz, https://norgeskart.no, www.ign.es/iberpix/visor/); encyclopaedias (www.wikipedia.org; www.britannica.com); online gazetteers and reference sites (e.g. www.wikidata.org, www.geonames.org (GeoNames), www.mindat.org); mountain classification systems (e.g. the International Standardized Mountain Subdivision of the Alps or SOIUSA for the Alps^22^, Alpenvereinseinteilung der Ostalpen^23^, Classification of the Himalaya^24^, www.peakbagger.com/rangindx.aspx (PEMRACS), www.carpathian-research-network.eu/ogulist, http://www.sopsr.sk/symfony-bioregio/lkpcarporog, www.dinarskogorje.com, https://bivouac.com/, https://climbnz.org.nz/); and national or regional landscape, geomorphological, or physiographic maps and publications^4,25–42^. The full list of the consulted sources and references is available on GitHub at https://www.github.com/GMBA-biodiversity/Inventory (GMBA Mountain Inventory v2.0 References.pdf).

All identified mountain ranges were recorded in a Microsoft Access relational database (“Mountain database”, see below) and given a name, a unique 5-digit identifier (GMBA_V2_ID), and the corresponding Wikidata unique resource identifier (URI), when available. This URI gives access to a range’s name as well as to its Wikipedia page URL in all available languages and lists other identifiers for given mountain ranges in a variety of other repositories such as GeoNames or PEMRACS. The primary mountain range names were based on the resources used for range identification and were preferably recorded in English. Names used nationally, locally, as well as/or by indigenous people and local communities were extracted from Wikidata and recorded in a separate attribute field.

In the process of cataloguing, we attributed a parent range to each of the mapped mountain ranges. Information about parent ranges is included in PEMRACS, often also in Wikidata as a property that can be extracted though a SPARQL query, in the corresponding Wikipedia pages description, and in regional hierarchical mountain classifications that exist for the European Alps (SOIUSA), the Carpathians, and the Dinaric Alps. When no such information was available, we relied on other sources of information that we found either using a general web search (leading to specific papers, reports, or web pages on mountain ranges) or by consulting (online) topographical maps and atlases at different scales. The information about parent ranges was used to construct a hierarchy of up to 10 levels using a recursive SQL query (see Step v). The result of this step was a relational database with a hierarchy of mountain systems and (sub-) ranges (Fig. 1, “Mountain database”).

### Step ii: Digitization of the mountain ranges

In a second step, we digitized all identified ‘childless’ mountain ranges (i.e. smallest mapping units, called ‘Basic’ as opposed to ‘Aggregated’ in the database) in one vector GIS layer. To do so, we used the Google Maps Terrain layers (Google, n.d.) as background and the WHYMAP named rivers layer^42^ as spatial reference since descriptions of mountain range areal extension is often given with reference to major rivers. The digitization, which was done in QGIS^43^ using the WGS 84 / Pseudo-Mercator (EPSG 3857) coordinate reference system, consisted in the drawing of shapes (polygons) that roughly followed the core area of each mountain range. In general, the approximate shape and extent of the mountain ranges we digitized could be distinguished based on the terrain structure as represented by the shaded relief background that corresponded to the placement and orientation of the range’s name label on a topographical map, atlas or other resource. As the exact placement and orientation of mountain range labels in each specific source can be influenced by cartographic considerations (e.g. avoiding overlaps with other features), the final approximation of the mountain range was obtained by consulting a variety of sources for each mountain range. Occasionally, the mountain terrain’s geomorphological characteristics strongly hampered the accuracy of our visual identification of mountain subranges within larger systems. This was particularly the case in old, eroded massifs such as the Brazilian Highlands or the highlands of Madagascar, where individual mountain ranges are not separated by deep well-defined valleys and have a very complex topography. In these cases, we referred to available topographical descriptions of range extent and to the river layer (see above). Other complex regions included Borneo and the Angolan Highlands, whereas subranges in mountain systems such as the European Alps, the Himalayas, and the North American Cordillera were comparatively easy to map. Moreover, the density of currently available mountain toponymical information varied quite strongly between regions. Accordingly, regional variation in the size of the smallest mountain range map units can be considerable. The result of this step was a (manually) digitized vector layer of named mountain ranges shapes (Fig. 1, “Manual mountain shapes”).

### Step iii: Definition of mountainous terrain

**Fig. 2:**
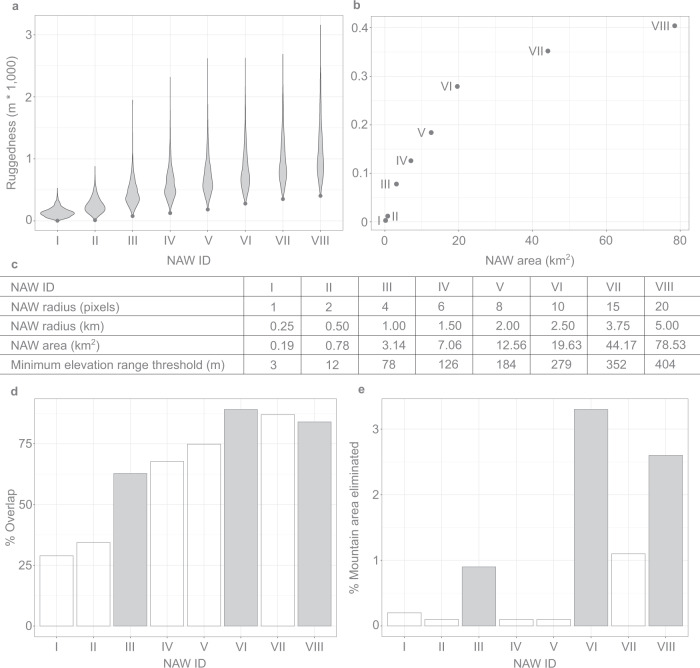
Elevation range thresholds for the eight neighbourhood analysis windows (NAW) and their contribution to calculations of the *GMBA Definition v2.0*. (**a**) distribution of elevation range values (ruggedness) for NAWs (numbered I to VIII) in mountain regions as defined by the geometric intersection of K1, K2 and K3. (**b**): plot of the minimum elevation range versus the area of the NAW (n = 920). (**c**) NAWs and their corresponding threshold values. (**d**) percent overlap between *GMBA Definition v2.0* (intersection of eight NAW-threshold pairs) and area defined by each individual NAW-threshold pair. (**e**) percent eliminated by each NAW-threshold pair (I to VIII) from the mountain area defined by the other 7 NAW-threshold combinations. Highlighted bars in the two graphs represent the combination of three NAW-threshold pairs that results in the highest overlap with the *GMBA Definition v2.0*.

In a third step, we defined mountainous terrain (*GMBA Definition v2.0*). To distinguish mountainous from non-mountainous terrain, we developed a simple algorithm which we implemented in ArcMap 10.7.1^44^. This algorithm is based on ruggedness (defined as highest minus lowest elevation in meter) within eight circular neighbourhood analysis windows (NAWs) of different sizes (from 1 pixel (≈ 250 m) to 20 (≈ 5 km) around each point, Fig. 2c) combined with empirically derived thresholds for each NAW (Fig. 2). The decision to use multiple NAW sizes was made because calculating ruggedness based on only a small or a large NAW comes at the risk of identifying the many local irregularities typically occurring in flat or rolling terrain as mountainous or of including extensive flat ‘skirts’ through the smoothing and generalization of large NAWs^3^. Accordingly, our approach ensures that any point in the landscape classified as mountainous showed some level of ruggedness not only at one but across scales. This also resulted in a smooth and homogeneous delineation of mountainous terrain, very suitable for our mapping purpose.

We used the median value of the 7.5 arc second GMTED2010 DEM^45^ as our source map. To reduce the latitudinal distortion of the raster, and thus the shape and area of the NAWs, we divided the global DEM into three raster layers corresponding to three latitudinal zones (84° N to 30° N, 30° N to 30° S and 30° S to 56° S) excluding ice-covered Antarctica and projected the two high latitude zones to Lambert Azimuthal Equal Area and the equatorial zone to WGS 1984 Cylindrical Equal Area. We used these reprojected DEM layers to produce eight ruggedness layers, each using one of the eight NAWs.

To determine the threshold values of our algorithm, we selected 1000 random points within the area defined by the geometric intersection (Fig. 1b) of the three commonly applied mountain definitions, i.e. the definitions by UNEP-WCMC^46^, GMBA^15^, and USGS^3^. These layers (referred to as K1, K2, and K3, respectively by Sayre and co-authors^12^) were obtained from the Global Mountain Explorer^47^. We eliminated 80 clearly misclassified points (i.e., points that fell within lakes, oceans, or clearly flat areas according to the shaded relief map we used as a background) and used the remaining 920 to sample the eight ruggedness layers. For each of the 8 layers, we retained the lowest of the 920 ruggedness values as the threshold for the layer’s specific NAW (Fig. 2c). The eight threshold values were then used to reclassify each of the eight layers by attributing the value 1 to all cells with a ruggedness value higher than or equal to the corresponding threshold and the value 0 to all other cells. Finally, we performed a geometric intersection (see Fig. 1b) of the eight reclassified layers to derive the new mountain definition.

After these calculations, we reprojected the three raster layers to WGS84 and combined them through *mosaic to new raster*. To eliminate isolated cells and jagged borders, we then generalized the resulting raster map by passing a majority filter (3 × 3 pixels, majority threshold) three times. This layer corresponds to the *GMBA Definition v2.0*.

The resulting mountain definition (*GMBA Definition v2.0*) distinguishes itself from previous ones because of the empirically derived thresholds method used to develop it and the use of eight NAWs. In line with the previous GMBA definition, it relies entirely on the ruggedness values within NAWs. The *GMBA Definition v2.0* was used to determine the outer delineation of this inventory’s mountainous terrain. As expected, it includes neither the wide ‘skirts’ of flat or undulating land around mountain ranges nor the topographical irregularities that are both typically included when other approaches are applied. It also successfully excludes extensive areas of rolling non-mountainous terrain such as the 52,000 km^2^ Badain Jaran Desert sand dunes in China. However, this mountain definition is conservative and only includes the highest, most rugged cores of low mountain systems, as for example in the Central Uplands of Germany, and therefore excludes some lower hill areas still considered by some as mountains.

As a further step towards generalization, we considered that small (<100 km^2^) inner-mountain flat areas corresponding to valley floors, small depressions, and isolated high plateaus were part of the mountainous terrain. Additionally, to avoid self-intersecting polygons in the final product we also eliminated mountain ‘appendages’ consisting of isolated raster cells smaller than 2 km^2^ and touching the main mountain area through one corner only. For the generalization process, we used a vectorized version of the mountain definition that we reconverted to a raster file for use in the creation of the *GMBA Inventory v2.0_standard* shapes in step iv. This simplification of the *GMBA Definition v2.0* was deemed necessary to generate the cleanest possible range shapes. Despite this generalization, we consider that these shapes can be used for most comparative mountain studies. However, for very precise area calculations, the new inventory layer can be intersected with the *GMBA Definition v2.0*.

### Step iv: Refinement of the ranges’ inner borders

To generate the final shape layer, we extended the hand-drawn polygons to the nearest surrounding river by allocating the value of the GMBA_V2_ID to all intermediate raster cells using the ArcMap tool ‘Cost Allocation’. For this, we intersected the simplified *GMBA Definition v2.0* (output step iii) with a rasterized river layer from Hydrosheds^2^. This resulted in a mask layer (Fig. 1, “Mask (GMBA)”) with value 1 for areas that are mountainous and not rivers and ‘NoData’ for non-mountain areas or rivers. We then combined this mask with the digitized vector layer of named ranges (Fig. 1 “Manual mountain shapes”, output step ii) and allocated to each cell the 5-digit GMBA_V2_ID value of the nearest digitized mountain range shape. In the allocation, rivers and non-mountain areas (value ‘NoData’ in the mask) acted as barriers to the cost allocation. This resulted in individual mountain ranges separated by rivers inside the overall *GMBA Definition v2.0* area. As the river cells maintained a value ‘NoData’ during the cost allocation operation, we performed a second cost allocation with a mask consisting of the *GMBA Definition v2.0* only, to fill these (river) cells with the nearest GMBA_V2_ID. Finally, we converted the resulting raster map to shapes representing the smallest mountain map units (‘Basic’ unit) of our inventory (Fig. 1, “Processed mountain shapes”).

### Step v: Preparation of the final products

**Fig. 3:**
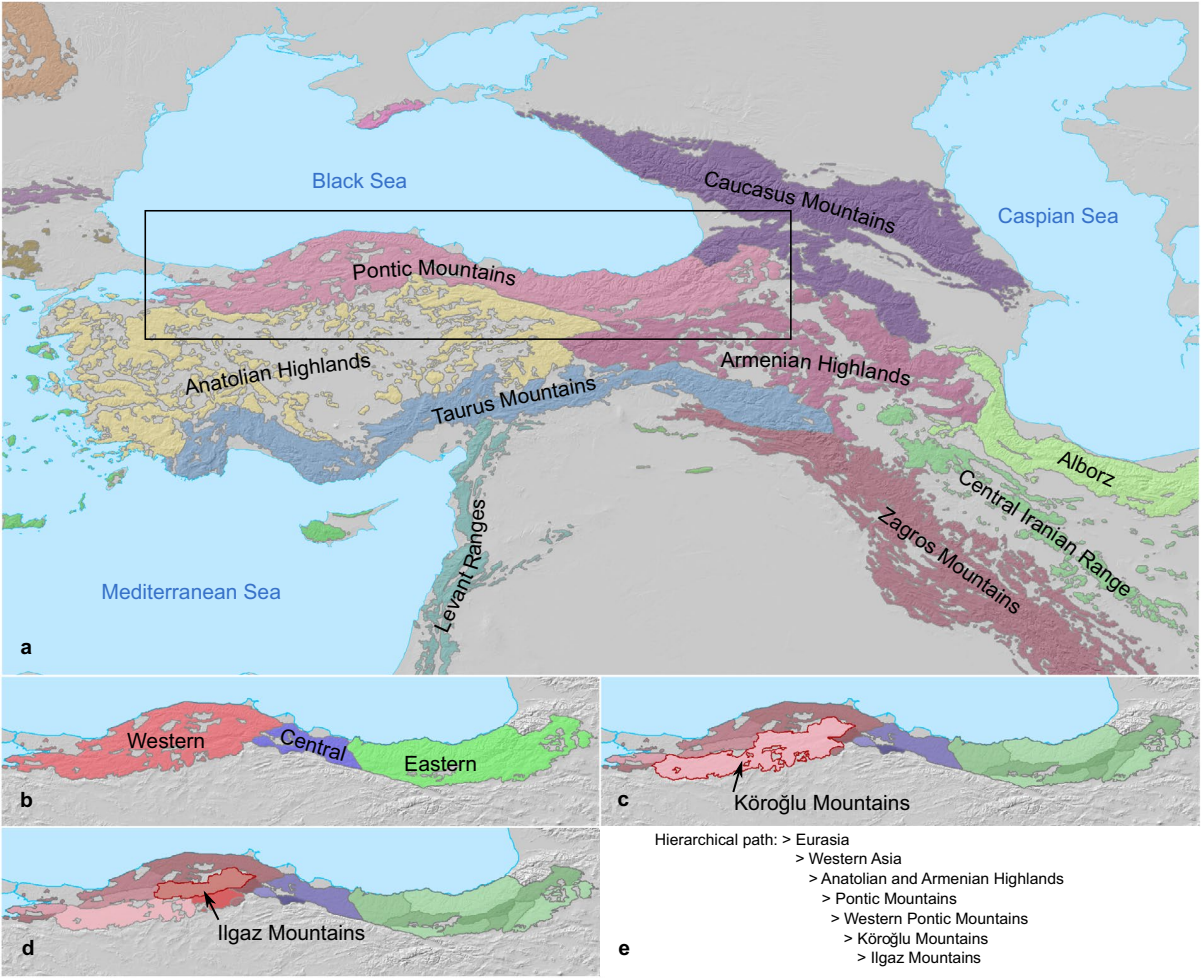
Illustration of the structure of the *GMBA Mountain Inventory v2.0_standard*. (**a**) section of the *GMBA Mountain Inventory v2.0_standard* for Anatolia, showing the major mountain systems and their smallest subdivisions. (**b–d**) levels 5 to 7 subdivisions of the Pontic Mountains. (**e**) hierarchical path leading to the Ilgaz Mountains, a sub-range of the Western Pontic Mountains highlighted in red in (**d**).

To create the final products, we first developed a recursive query to convert the parent-child relations recorded in the “Mountain database” (output step i) into unique hierarchical paths (see Fig. 3e), leading from the basic mapping unit up to the highest level of aggregation (Level 1, continents and oceans). We then combined the mountain range shapefile layer (“Processed mountain shapes”, output step iv) with an export query of the “Mountain database” as an attribute table containing the complete hierarchy for each mapped mountain range. This allowed us to construct all the higher parent ranges levels by dissolving according to individual levels in the hierarchy. The resulting layer representing mountain range shapes at the ten levels in the hierarchy were merged into one final ‘stacked’ shapefile entitled *GMBA Inventory v2.0_standard* containing all mountain range shapes at all (overlapping) hierarchical levels (Fig. 3).

To produce a mountain inventory that can be intersected with any of the three mountain definitions currently in use and available on the Global Mountain Explorer, we also applied the ‘cost allocation’ (step iv) to an additional, considerably broader mountain layer that we obtained in step (iii) by geometric union (Fig. 1c) of the UNEP-WCMC, GMBA, and USGS layers. A 5 km buffer was added to ensure that small, isolated mountain patches would be connected and thus facilitated the cost allocation procedure. We then processed the resulting layer (“Broad”) following the exact same steps (iv and v) as above to generate a second version of the raster map and a second version of the ‘stacked’ shapefile. This second version, entitled *GMBA Inventory v2.0_broad*, enables comparative mountain science based on a different definition of mountains than the one presented here but needs to be intersected with the chosen definition before use.

## Data Records

### New GMBA Definition v2.0

The *GMBA Definition v2.0.tif* is a high resolution (7.5 arcsecond) binary raster file identifying areas considered mountainous with the value 1 and non-mountain areas with the value ‘NoData’.

### GMBA Inventory v2.0_standard

The *GMBA Inventory v2.0_standard*.*shp* (Table 1) is a ‘stacked’ shapefile including the 8327 shapes of the inventory (at all hierarchical levels) that overlaps with the *GMBA Definition v2.0*. These shapes include both *basic* map units (i.e. units without ‘child’ sub-division at the bottom of the hierarchy) and *aggregated* ones (i.e. ‘parent’ ranges higher up in the hierarchy). In this inventory, the outer borders of the mountain ranges and systems (i.e., the borders between mountainous terrain and surrounding flat or rolling land) correspond to the newly proposed *GMBA Definition v2.0*. Because all parent and child polygons are included in this layer, there is a high level of overlap (up to ten overlapping polygons for the few cases where the hierarchy consists of all 10 levels).

**Table 1:** Data attributes of *GMBA Inventory v2.0_standard* and *GMBA Inventory v2.0_broad*.

Attributes	Objects/file size
**GMBA_V2_ID**: A five-digit unique identifier of the mountain range, linking the polygon in the shapefile with the record in the “Mountain database”.	***GMBA Inventory v2.0_standard*** Type: ESRI shapefile 8327 partly overlapping polygons representing the spatial extent of all the polygons (across all hierarchical levels) included in the inventory File size: 322 mB ***GMBA Inventory v2.0_broad*** Type: ESRI shapefile 8616 partly overlapping polygons representing the spatial extent of all the polygons (across all hierarchical levels) included in the inventory File size: 252 mB
**GMBA_V1_ID**: The ID of the polygon in the *GMBA Inventory v1.0*.	
**MapName**: Mountain range name that can be used for labelling the polygons on a map (duplicates exist for different mountain ranges sharing the same name).	
**WikiDataUR**: A unique resource identifier (URI) starting with Q, identifying the mountain range in the Wikidata repository. This URI can be used to retrieve data related to the feature from Wikidata (this includes, for example, the name in various languages and the Wikipedia URL in various language versions).	
**MapUnit**: Categorical variable (2 unique values): ‘Basic’ represents all polygons that are basic mapping units, i.e. they have no child. Selecting polygons identified as ‘Basic’ returns all the smallest mountain polygons in the inventory. ‘Aggregate’ identifies those polygons that have children and are thus aggregations of ‘Basic’ polygons.	
**Hier_Lvl**: Identifies the level of each polygon in the hierarchy with a number from 1 to 10. This can be used to disaggregate the layer into its ten hierarchical components.	
**Feature**: Categorical variable (14 unique values) indicating what the polygon represents, see Table 1.	
**Area**: Calculated planimetric area of the mountain polygon (in km^2^) (calculated in Mollweide projection). Not available for *GMBA Inventory v2.0_ broad*	
**Perimeter**: Calculated perimeter of the mountain polygon (in km). Not available for *GMBA Inventory v2.0_ broad*.	
**Elev_Low**: Lowest elevation calculated from GMTED 2010 7.5 arcsecond DEM (can include negative values, e.g. in mountains bordering the Dead Sea).	
**Elev_High**: Highest elevation as calculated from GMTED 2010 7.5 arcsecond DEM.	
**Path**: The full hierarchical path leading to the mountain range (using DBaseName) starting at level 1 (continents/oceans).	
**PathID**: The full hierarchical path leading to the mountain range expressed as a concatenation of GMBA_V2_IDs	
**Level_01 to Level_10**: Ten columns each containing the hierarchical levels for each mountain range. These are the same levels as in “Path”, but one level per column.	
**Select_300**: A customised selection of 292 mountain ranges / systems, useful for global and IPCC or IPBES (sub-) regional level analyses. It was generated by using the GMBA Inventory v2.0_SelectionTool.	
**Countries**: String variable with country names that intersect with a mountain range.	
**CountryCodes**: String variable with Alpha-3 ISO country codes (https://en.wikipedia.org/wiki/ISO_3166-1_alpha-3) that intersect with a mountain range.	
**DBaseName**: Unique mountain range name for identification in the database. For example, there are many ranges called ‘Black Mountains’ that all have the same name in MapName, but are identified in DBaseName as ‘Black Mountains (New Zealand)’, ‘Black Mountains (USA)’ etc. The indication ‘(nn)’ after a range name, means that this polygon represents a subrange that has no name yet.	
**LocalNames**: Wikidata names of the mountain range or feature in all languages spoken in the countries where the mountain range occurs.	
**AsciiName**: MapName without diacritical marks.	
**Name_xx**: Range name in Arabic, Chinese, English, French, German, Portuguese, Russian and Spanish.	
**ColorAll**: A number from 1 to 6 to for colouring polygons with six distinct colours without any contiguous shape sharing the same colour. For use with the ‘Level’ layers.	
**ColorBasic**: idem, for use with the ‘Basic’ layer.	
**Color300**: idem, for use with the ‘300’ layer	

Besides mountain systems, ranges, and subranges, the 8327 mapped units in the *GMBA Inventory v2.0_standard* consist of different types of geographical features (‘Feature’ in Table 2), including islands, archipelagos, peninsulas, highlands, plateaus, and escarpments. These feature categories were broadly taken from PEMRACS and were included to ensure the largest possible coverage of mountainous physiographical elements at different scales/levels, even if no mountain range name was found. This is particularly the case for mountainous islands, which are clearly defined spatial objects requiring a place in the hierarchy. In the case of spatial features such as islands, a polygon named after the given feature (e.g., Sardinia) represents the mountainous area of that feature, not the entire island. It could therefore also be named ‘Sardinian mountains’ or ‘Sardinian Highlands’, but this has only been done if such a naming was accepted in literature, for example for the ‘New Guinea Highlands’. Commonly used geographic divisions of mountain systems, such as the Eastern Alps or Northern Andes, were also included in the hierarchy and identified as ‘Geographically-defined subrange’. So-called ‘support polygons’ were introduced either to close the gap between parts of named subranges within a range, or to introduce an intermediate level in the hierarchy for aggregation purposes. Some were given a name, if they corresponded to a well-defined region, while others were left with the name of the parent range and the addition ‘(nn)’ for ‘no name’. The various features (including support polygons) that do not strictly correspond to mountain systems, ranges, and subranges might be updated with a mountain range name in the future, when more toponymical resources become available, or if users provide specific feedback.

**Table 2:** Inventory statistics.

A. Feature	Number of mountain ranges by feature					
	*GMBA Inventory v2.0 standard*			*GMBA Inventory v2.0 broad*		
	Basic	Aggregated	Total	Basic	Aggregated	Total
Mountain range with well-recognized name	4953	682	5635	5113	684	5797
Geographically-defined sub-range	257	390	647	266	393	659
Support polygon - unnamed	539	0	539	553	0	553
Support polygon - named	279	204	483	294	209	503
Island	245	65	310	287	66	353
Highland or plateau	114	93	207	127	95	222
Miscellaneous physical or political feature	89	51	140	96	52	148
Archipelago	45	84	129	48	85	133
Area dominated by a single large peak	90	7	97	91	7	98
Peninsula	40	16	56	46	16	62
Point Defined Range	53	0	53	56	0	56
Escarpment/Canyon	13	2	15	14	2	16
(Sub)-Continent	0	12	12	0	12	12
Ocean	0	4	4	0	4	4
Grand Total	6717	1610	8327	6991	1625	8616
**B. Hierarchical level**	**Number of mountain ranges by hierarchical level**					
	***GMBA Inventory v2.0 standard***			***GMBA Inventory v2.0 broad***		
	**Basic**	**Aggregated**	**Total**	**Basic**	**Aggregated**	**Total**
1	0	9	9	0	9	9
2	5	41	46	5	41	46
3	22	156	178	29	159	188
4	529	346	875	582	350	932
5	1361	494	1855	1443	498	1941
6	2464	411	2875	2517	411	2928
7	1737	125	1862	1777	129	1906
8	476	25	501	506	25	531
9	116	3	119	125	3	128
10	7	0	7	7	0	7
Total	6717	1610	8327	6991	1625	8616

Number of mountain range shapes according to (A) feature type and (B) level in the hierarchy (level 1 is the highest (continent) and level 10 is the lowest (smallest sub-range)). Results by map unit (basic vs. aggregated) and map version (*GMBA Inventory v2.0_standard* vs *GMBA Inventory v2.0_broad*).

### GMBA Inventory v2.0_broad

The *GMBA Inventory v2.0_broad*.*shp* layer (Table 1) is similar to the *GMBA Inventory v2.0_standard* shapefile, but the outer borders correspond to the “Broad” layer (see step v). Because the mountain extent is considerably larger in the “Broad” than in the *GMBA Definition v2.0*, it also includes more of the mountain ranges identified in the “Mountain database” and the “Mountain manual shapes” layer. Accordingly, with 8616 shapes, this inventory includes 289 more named ranges (at all hierarchical levels). As it includes the terrain that fulfils the criteria of all three mountain definitions, this broad version of the inventory also includes high plateaux such as those of the Andes (*altiplano*) and Tibet, provided they reach above 2500 m.a.s.l.

### GMBA Inventory v2.0_Selection_Tool and gmbaR R package

The *GMBA Inventory v2.0_Selection_Tool*.*xlsx* is a spreadsheet (MS Excel workbook, Table 3), in which the rows represent all 8616 mountain ranges and their position in the hierarchy, and which includes programmed functions to enable customized selections of mountain ranges at intermediate levels of aggregation. It can be used independently or in combination with the R package *gmbaR* (see Code Availability) to make customized selections of mountain ranges (see Usage Notes).

**Table 3:** Data attributes of *GMBA Inventory v2.0_Selection_Tool*.

Attributes	Objects / file size
**GMBA_V2_ID**: See Table 1.	***GMBA Inventory v2.0_SelectionTool.xlsx*** Type: Excel workbook File size: 1.5 mB
**GMBA_V1_ID**: See Table 1.	
**MapName**: See Table 1.	
**DBaseName**: See Table 1.	
**Path**: See Table 1 (hidden by default).	
**Level01 to Level10**: Mountain range in path structure. This includes all the levels leading to the mountain range. It is used for the worksheet functions. These columns are hidden by default.	
**Level_1 to Level_10**: Mountain range in hierarchical structure. This version of the levels only shows the mountain range name in its hierarchical position. This is mainly a visual difference that better displays the hierarchical structure.	
**Range_Selector**: The column where mountain ranges can be selected by adding an “x” in the corresponding row.	
**Selected_Range**: A formula column that fills the selected range for all its child ranges.	
**Level_Selected**: A formula column that fills the hierarchical level of the selected range for all its child ranges.	
**GMBA_V2_ID_Selected**: A formula field that returns the GMBA_V2_ID for the selected range.	
**Overlap_Warning**: A formula field which returns the message “polygons overlap!” if a subrange of a selected parent range is selected.	
**GMBA_V2_ID_DissolveField:** A formula field which returns the GMBA_V2_ID of the corresponding selected parent range. It can be used in combination with the GMBA_V2_ID of the range, exported to txt or csv, and used as a dissolve field to construct the polygons.	
**MapUnit**: See Table 1.	
**Hier_Lvl**: See Table 1.	
**Feature**: See Table 1.	
**Area**: See Table 1.	
**Perimeter**: See Table 1.	
**Elev_Low**: See Table 1.	
**Elev_High**: See Table 1.	
**Elev_Range:** Elevation Range (Elev_High minus Elev_Low).	
**Lat_Centr:** Latitude of the mountain range centroid.	
**Lon_Centr:** Longitude of the mountain range centroid.	
**Select_300**: See Table 1.	
**Standard:** Identifies those ranges that appear in “GMBA Inventory v2.0_standard”.	
**Countries**: See Table 1.	
**IPCC_maj**: IPCC AR6 subregion with the largest overlap with the mountain range.	
**IPCC_str**: List of all IPCC AR6 regions intersecting with the range.	
**IPBES_maj**: IPBES subregion with the largest overlap with the mountain range.	
**IPBES_str**: List of all IPBES subregions that intersect with the mountain range.	
**AsciiName**: See Table 1.	
**Name_xx**: See Table 1.	

The *GMBA Definition v2.0* the *GMBA Inventory v2.0_standard*, and the *GMBA Inventory v2.0_broad* are available for unrestricted download on the GMBA Mountain Inventory page^18^ of EarthEnv. The two inventory versions can be seamlessly read into R using gmba_read() from *gmbaR*. The *GMBA Inventory v2.0_Selection_Tool* is available on GitHub at https://www.github.com/GMBA-biodiversity/Inventory (*GMBA_Inventory_v2.0_Selection_Tool.xlsx*).

## Technical Validation

To validate the inventory, we performed a consistency check regarding mountain range names and extents, and an evaluation of the completeness of the recorded mountain ranges. The validation of the new mountain definition (*GMBA Definition v2.0*) consisted in a quantification of the contribution of the selected NAWs in capturing mountain terrain and a comparison of the new mountain definition with the three definitions featured on the Global Mountain Explorer.

### Mountain inventory

#### Mountain range names and extents

Names and extents of mountain ranges were validated during the collation process by consulting several sources for each entry (see step i). Besides occasional differences, the naming and geographical extent of mountain ranges showed a high level of consistency between sources. However, mountain ranges are typically known under different names through space (e.g. north or south of the range) and time (for example when colonial names superseded the original indigenous ones)^48^. To account for this diversity, we queried Wikidata for alternative local and indigenous names and added these to the database (LocalNames, Table 1). In general, the consistency in naming and extent of mountain ranges was highest for smaller mountain ranges and well-defined, isolated large mountain systems (such as the European Alps and the Andes). In contrast, the naming and extent of larger mountain ranges within mountain systems such as the Kun Lun Mountains, the Hengduan Mountains, and their subranges could differ quite substantially between sources. Here, we adopted a conservative approach, attributing the smallest number of reported subranges to each mountain system.

#### Completeness of the inventory

To evaluate the completeness of the inventory, we calculated the percentage of mountain range names gathered from two reference lists that are also included in the former (GMBA Inventory v1.4) and current (*GMBA Inventory v2.0_standard*) inventory, respectively (Table 4).

**Table 4:** Validation of the inventory.

	Unique names	All names
	**WoS list**	
GMBA Inventory v1.0	**40.9**% (113/276)	**48.1**% (252/524)
*GMBA Inventory v2.0_standard*	**91.7**% (253/276)	**95.6**% (501/524)
	**GMBA member list**	
GMBA Inventory v1.0	**36.7**% (84/229)	**52.7**% (432/819)
*GMBA Inventory v2.0_standard*	**90.8**% (208/229)	**96**% (786/819)

Validation against a selection of mountain ranges extracted from the Web of Science and from the GMBA member database. The column ‘Unique names’ gives the number of distinct ranges reported, while the column ‘All names’ includes repetitions of the same ranges.

To generate the first reference list (WoS list) we first queried the Web of Science with the keyword string “mountains” OR “mountain range” NOT “mountain pass”, sorted the obtained publications by relevance, selected those published in 2020, and exported the first 500 records. We then manually extracted the mountain range names from the title, abstract, and keywords fields, or searched in the methods section (study site) if no mountain range name was mentioned in these fields. We extracted 524 range names, of which 276 were unique. The second list (GMBA member list, which includes mountain scientists from 69 countries across the world) consisted of all the mountain ranges named by GMBA members as their (geographical) area of work and/or expertise. We extracted 819 mountain range names, of which 229 were unique. For both lists, we individually checked all mountain range names for differences in spelling or alternative names.

We calculated the percentage both for unique names (i.e., each unique mountain range name is counted only once) and for all names (i.e., including repetitions). The latter value better captures the likelihood that a mountain range used in the academic context is represented in the inventory.

The completeness percentages calculated based on the WoS and GMBA member lists show a high level of agreement. Based on our samples, the likelihood that a mountain range reported in the academic literature is also included in our new inventory is higher than 95% (Table 4). This likelihood is about two times higher than the likelihood of inclusion in the previous inventory (GMBA Inventory v1.4).

The results of this completeness analysis do not inform about the absolute completeness of the *GMBA Inventory v2.0*, as many and especially smaller ranges are not (yet) included. These results rather indicate that most of the mountain ranges or mountain systems generally referred to in the academic literature and by the mountain research community are well represented in the inventory. Compared with all objects classified as mountain range in the Geonames gazetteer (category “MTS”: 26,312 entries on 22 March 2021) and in Wikidata (category “Q46831” mountain range: 20,768 entries on 22 March 2021), the completeness of our inventory is modest, but these repositories include many very small mountain ranges as well as many double entries (Wikidata).

From the 1033 polygons common to both versions of the GMBA Inventory, 759 have remained ‘basic’ map units (i.e., they were not attributed any child ranges), while 274 polygons were split into subranges in the *GMBA Inventory v2.0_standard*.

### Mountain definition

#### Sensitivity analysis of the NAW-threshold pairs

All global geomorphometric approaches to mountain definitions presented on the Global Mountain Explorer calculate slope or elevation range (ruggedness) metrics using unique combinations of input DEM (with their respective cell size), NAW number and size, and threshold value(s)^8,12^. In all cases, the size and shape of the NAWs as well as their corresponding thresholds for a given cell size of the elevation raster (DEM) are based on expert judgement. Here, we derive parameters for the NAW and their corresponding threshold values empirically by sampling the 920 random points (see step iii in Methods) in an area considered as mountainous by all three definitions featured on the Global Mountain Explorer.

For validation purposes, we assessed the relative contribution of the different NAW-threshold pairs to the final mountain definition. For attributing the value ‘mountainous’ to a cell, the algorithm requires the local elevation range calculated for each NAW to be higher than its corresponding threshold value. Because of this, each additional NAW-threshold pair reduces the area considered as mountainous and thus contributes towards spatially refining the final mountain area by intersection (see Fig. 1b).

We first calculated the percentage correspondence between the area identified as mountainous according to each NAW-threshold pair and the final mountain definition. The larger this percentage, the better the NAW-threshold pair captured the mountain terrain (Fig. 2d). We also calculated the percent difference in mountain terrain between the final mountain definition and the mountain area achieved when leaving out one of the eight NAW-threshold pairs from the algorithm at a time. The larger the percentage area, the more important the corresponding NAW-threshold pair was for refining the mountainous terrain area (Fig. 2e). Finally, we calculated the difference between the mountain definition and all combinations of two and three NAW-threshold pairs.

When assessing the relative contribution of each NAW-threshold pair to the final mountain definition (Fig. 2d) we observed a steady increase from small to larger NAWs, with a peak at the 10 pixel NAW (5 km diameter), which overlaps 89% with the final mountain terrain. When looking at combinations of two NAWs, the combination of the 10 and 20 pixel NAW comes closest with 95.6% overlap. This overlap increases to 98% after adding the 4 pixel NAW.

As expected, the smaller NAWs and their empirically derived thresholds were least effective in delimiting mountainous terrain because these ruggedness thresholds are too low to adequately capture small areas of less rugged terrain (valley floors, gentle lower slopes etc) also included in mountain areas. Applying the two smallest NAWs only marginally improves the definition by allowing the elimination of only 0.1 to 0.2% from the total mountain area (Fig. 2e), while, when used in isolation, these NAW-threshold-pairs include up to 70% non-mountainous area. If these smaller NAWs were used alone (i.e., not in combination with some “smoothing” larger NAWs), and with a higher corresponding threshold, the result would be considerably patchier, including many small mounds in flat or rolling terrain, and many small “flat” areas in mountainous terrain.

When comparing the current algorithm with the original NAW-threshold pair applied in Körner *et al*.^10,15^ (mean NAW size = 3.4 km^2^, threshold > = 200 m), we see that at a similar maximum distance within the NAW (around 3 km), the current algorithm applies a lower threshold value (126 m, see Fig. 2c). This is to be expected as the scale-dependent choice of different NAW-threshold pairs in the current algorithm allows for each NAW-threshold pair to be less restrictive than in the case of a unique NAW-200 m ruggedness threshold pair.

#### Comparison of mountain definitions

To compare our refined mountain definition with existing ones, we calculated and compared their planimetric area.

**Fig. 4:**
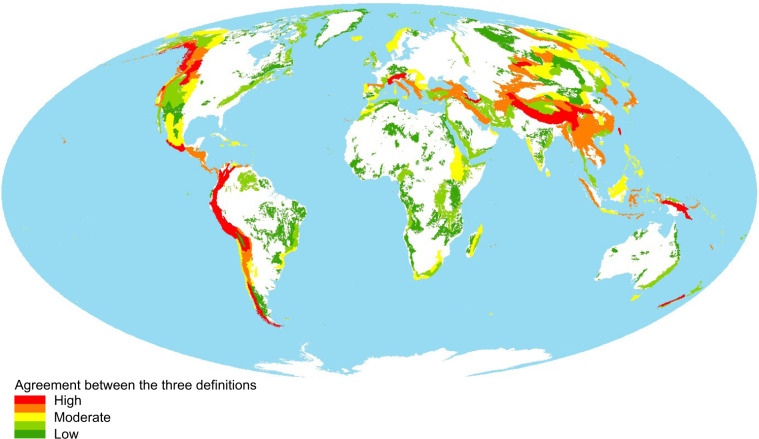
Level of agreement between the different mountain definitions. The map shows 292 major mountain ranges/systems and the values represent the smallest area of mountain area according to any of the 3 definitions expressed as a percentage of the total area for each mountain range (union of all definitions). Greens: low level of agreement, reds: high level agreement. The Mollweide projection was used to preserve areas.

Table 5 shows that the overall mountain area according to the *GMBA Definition v2.0* applied is about 50% larger than that estimated based on the previous GMBA mountain definition (GMBA Definition v1)^10,15^ as a result of the smoothing effect of the new algorithm (see Methods). With a coverage of about 18.2% of the global land area excluding Antarctica, this new mountain definition lies between the GMBA Definition v1 (12.3%) and the definitions proposed by UNEP-WCMC (24.2%) and USGS (30.4%). However, these differences in mountain cover according to the different definitions are not homogeneously distributed across regions. In major, large, high, and rugged mountain systems (e.g. the Andes, the European Alps, the Caucasus, the Dinaric Alps, the Pindus, the Himalayas and associated mountain systems, or regions such as the spines of New Guinea, Sumatra, and the Japanese Archipelago), the correspondence between areas classified as mountainous according to the various definitions is much higher than in lower, less well-defined and less rugged mountain systems (e.g. Brazilian Highlands, highlands of Madagascar, or Central European Highlands, see Fig. 4), where the WCMC and USGS definitions tend to include much more rolling land and wider flat skirts than the more conservative GMBA definitions.

**Table 5:** Planimetric area and percent coverage of mountain terrain according to the current approach and previous ones (GMBA Definition v1, UNEP-WCMC, and USGS).

	GMBA v1 (2m30s)	GMBA v2 (7.5 s)	UNEP-WCMC (30 s)	USGS (7.5 s)
	Körner *et al*. 2011 (K2)	Snethlage *et al.* 2022	Kapos *et al*. 2000 (K1)	Karagulle *et al*. 2017 (K3)
Area (Mio. km^2^)	16.54	24.53	32.65	40.93
Cover (%)	12.28	18.21	24.23	30.38
Cover as % of GMBA Definition v2.0	67.43	100.00	133.10	166.86

For each definition, the value between brackets indicates the resolution of the elevation raster in arc minutes and seconds. The *GMBA Definition v2.0* layer corresponds to the output of step iii after the application of the majority filter. The columns are not ordered by year of publication of the mountain delineations but increasingly relative to the cover percentage of the *GMBA Definition v2.0*.

## Usage Notes

### Polygon extent

A key difference between the *GMBA Inventory v2.0_standard* and the *GMBA Inventory v2.0_broad* is the extent of the outer borders of the mapped mountain ranges. In the *GMBA Inventory v2.0_standard*, the shapes’ outer borders correspond to the mountain definition introduced in this paper (*GMBA Definition v2.0*). In the *GMBA Inventory v2.0_broad*, outer borders correspond to the union of the three mountain definitions presented on the Global Mountain Explorer (Fig. 1c) and a 5 km buffer. Accordingly, users adopting this version first need to intersect it with the mountain definition of their choice (K1, K2 or K3, as found on the Global Mountain Explorer). The resulting raster layer can be used as such for analysis or converted to polygons (see “Checking geometry”).

### Checking geometry

The process of converting the mountain rasters to shapes (step iii) results in cases of apparent shape self-intersections, for example in points where shapes touch themselves. The layers provided have been geometrically corrected by applying a buffer of size zero, but it is prudent to check and correct the geometries before the shapes are used in any calculations, especially after intersecting the *GMBA Inventory v2.0_broad* version with one of the mountain definitions and polygonising it.

### Selecting polygons from the hierarchical structure

The hierarchical structure allows users to zoom into mountain systems and their subranges and explore the changing spatial patterns with increasing spatial resolution. However, as a result of differences in mountain toponymical information density and of physiographic differences in the position of mountain systems and their subranges, the number of levels in the mountain hierarchy constructed from the parent-child relationships for each basic unit varies between 4 and 10. This puts many small ocean island ranges at the same level in the hierarchy (level 4) as some continental mountain systems, which is not useful for global scale comparative mountain research. For such research, we encourage the use of our selection of 292 mountain systems or a custom selection of non-overlapping mountain ranges made using the *GMBA Inventory v2.0_SelectionTool* or the associated R package *gmbaR*.

The Excel-based Selection Tool (*GMBA Inventory v2.0_Selection_Tool.xlsx*) includes the entire *GMBA Inventory v2.0_standard* attribute table and allows the user to make a customised selection of mountain ranges. Changing the cell value in column ‘Range Selector’ to ‘x’, selects the range and all its subranges (the range and its subranges are highlighted). If a child of any selected range is also selected (i.e., a subrange within the highlighted list), a warning appears in column “Overlap_Warning”, as a parent always overlaps with a child. The list of selected mountain range IDs (GMBA_ID_V2) can be imported into a GIS or to R to select the desired mountain range shapes. Alternatively, the R package *gmbaR* allows seamless work with the mountain inventory in R (the package can be installed from https://github.com/GMBA-biodiversity/gmbaR). The *GMBA Inventory v2.0* can be directly read to R, and mountain ranges can be selected based on, for example, different Selection Tool attributes (gmba_select()) or point coordinates (gmba_ids_from_points()).

### Limitations

The *GMBA Inventory v2.0* is a spatially explicit inventory of mountain ranges across the world of unprecedented completeness. Several assumptions or decisions had to be made to make the development of this resource explicit and transparent:

i. Status of the inventory: The *GMBA Inventory v2.0_standard* is an operational and spatially explicit collection of mountain range identifiers at various geographical scales. It offers a listing of mountain range names and associated map shapes representing their spatial extent. It enables quantitative comparisons from a regional to the global level. The inventory does not replace existing national or regional mountain classifications nor does it pretend to represent a new standard or official gazetteer.
ii. Naming of the mountain ranges: for the primary mountain range identifier we have given preference to the name as it appears on topographical maps. However, other sources of mountain range identification (such as landscape and physiographic classifications or complete mountain hierarchies) sometimes use different nomenclature, and mountain ranges are sometimes known under different names according to the source or classification system. Given that in the (scientific) literature geographical features are typically named in English, we chose the English name as the main textual identifier of most map units in the inventory. In addition, we provide the names in seven globally spoken languages, as well as in all additional languages spoken in the countries where the range occurs and available on Wikidata. By doing so, we also include indigenous or local names. To date, the number of mountain ranges with indigenous or local language translations is still very limited in Wikidata.
iii. Delineation of the mountain ranges: no universal agreement exists as to where a mountain range grades into surrounding lower areas. Different attributes of landforms can be considered to determine what a mountain (range) is (relief, lithology, geomorphology, culture), and such perspectives are different between individuals and communities. Here we adopted a purely morphometric approach, which leads to a conservative delineation of low and scattered mountains, in which only the most rugged cores have been included. The shapes representing these mountain ranges in the inventory might therefore differ quite markedly from what is generally considered to be part of these low mountain systems. As the outer borders of the mountain shapes in the *GMBA Inventory v2.0_broad* correspond to the geometric union of the three definitions (and an additional 5 km buffer) it should only be used after intersection with one of these definitions available on the Global Mountain Explorer, or any other mountain definition.

## Data Availability

The R package gmbaR provides a set of R functions to read and work with the *GMBA Inventory 2.0*. This package is provided, explained, and continuously developed on Github (https://github.com/GMBA-biodiversity/gmbaR).
